# Innate lymphoid cells type 3 in cancer

**DOI:** 10.3389/fimmu.2022.1033252

**Published:** 2022-10-13

**Authors:** Raquel Castillo-González, Ana Valle-Noguera, Maria José Gomez-Sánchez, Pu Xia, Aranzazu Cruz-Adalia

**Affiliations:** ^1^ Pathology Anatomy Department, Instituto de Investigación Sanitaria Hospital 12 de Octubre (imas12), Madrid, Spain; ^2^ Universidad Autónoma de Madrid (UAM), Madrid, Spain; ^3^ Department of Immunology, Ophthalmology and Ear, Nose and Throat (ENT), Complutense University School of Medicine and Instituto de Investigación Sanitaria Hospital 12 de Octubre (imas12), Madrid, Spain; ^4^ National Center for Radiation Research in Oncology (OncoRay) - National Center for Radiation Research in Oncology, Faculty of Medicine, University Hospital Carl Gustav Carus, Helmholtz-Zentrum Dresden-Rossendorf, Technische Universität Dresden, Dresden, Germany

**Keywords:** ILC3, cancer, tumor microenvironment, IL-22, IL-17

## Abstract

Cancer is a multifactorial chronic illness caused by a combination of genetic and environmental factors. A tumor is more than just a collection of cancer cells, it also contains infiltrating and resident host cells that are constantly interacting with it. Innate lymphoid cells (ILCs) have been recently found to be within the tumor and its microenvironment in close relationship with cancer cells. Although ILCs lack an antigen-specific receptor, they can respond to environmental stress signals, aiding in the fast orchestration of an early immune response. They are tissue resident cells mostly located in mucosa and first barrier organs that have been mainly studied in the defense against pathogens, lymphoid development, and tissue repair, however, current research has begun to elucidate their involvement in carcinogenesis. Nevertheless, among all ILCs, ILC3s have been found to be the most controversial in terms of tumor immunity. It has been found that they enhance anti-tumor immunity by detecting cancerous cells and helping lymphocytes infiltrate tumors. However, some recent studies have revealed that IL-23 stimulating ILC3s may promote tumor growth. In this review, we have incorporated the most recent studies on the involvement of ILC3s in cancer development to offer an overview of the role of ILC3s in cancer emphasis on their particular activity in several organs primarily in the mucosa, but also in breast, pancreas, liver, and skin, realizing that their role likely depends on the tissue microenvironment and the subtype of ILC3s.

## Introduction

Cancer is a multifactorial and complex disease that can affect any organ of the body, thus making it one of the most difficult diseases to study and treat. The transformation outcome of malignant tissue is closely related to immune cells (1). Tumor microenvironment provides necessary support for tumor growth, invasion and metastasis (2). Within immune cells, innate immunity reacts by killing directly or indirectly abnormal mutant cells in the initial stage of tumor (3). One of the key players of innate immune cells are innate lymphoid cells (ILCs) that are characterized by lack of recombination activating gene (RAG) - dependent antigen receptor rearrangement and absence of T cell or B cell receptors (TCR\BCR) (4). They are divided into five categories, natural killer (NK) cells, ILC1s, ILC2s, ILC3s and lymphoid tissue inducer cells (LTi) cells (5, 6).

ILC3s and LTis express the transcription factor retinoid orphan receptor gamma t (RORγt), function similarly to Th17 cells and play an important role in the fight against extracellular microbial infections mostly in intestinal tissue (7, 8). RORγt^+^ ILC3 cells are abundant in intestinal tissue, and they play an important role in intestinal mucosal diseases and host defense (9). ILC3s are divided into two subpopulations, natural cytotoxicity triggering receptor 1 (NCR) NCR^+^ ILC3s and NCR^-^ ILC3s, by NKp44 (human) and NKp46 (mouse) expression (10) and they secrete mainly interleukin 17 (IL-17) and IL-22, which are involved in maintaining intestinal tissue balance and promoting intestinal stromal cell proliferation (11). The role of ILC3s in tumor immunity has been studied during the last few years, however their role is not clarified yet due to the controversy between the studies. There is a lack of knowledge about which ILC3 subsets and Lti are involved in the process, due to an inefficient characterization of specific markers. Moreover, the role could change depending on the tumor microenvironment developed in the different organs. The detailed relationship between ILC3s and various types of tumors will be shown in the following sections detailing the molecular mechanisms and if possible, the subtype of ILC3/Lti involved in the system.

## Gut cancer

Among gastrointestinal cancers, colorectal cancer (CRC) is the most prevalent and diagnosed in the United States (12), ranks third in terms of incidence and is the fourth most prevalent cause of cancer death in the world (13). The initiation and cause may be sporadic, hereditary, or related to inflammatory bowel disease (IBD). In the case of IBD, long periods of persistent inflammation occur causing increased intestinal epithelial cell (IEC) turnover, cell damage, dysplasia and genetic and immunological alterations in IECs which raises the risk of CRC in affected patients. Furthermore, some proinflammatory chemokines and cytokines secreted during flares have been demonstrated to rule tumorigenesis, for example, IL-17 and IL-22, which are secreted by ILC3s and T cells (14–16). However, the precise involvement of ILC3s in intestinal cancer development remains at present incompletely characterized.

**Figure 1:**
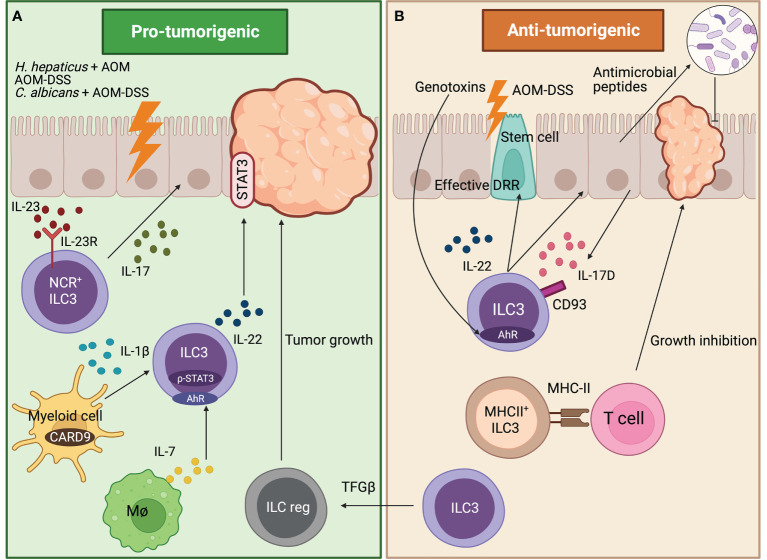
Role of ILC3s in tumors developed in the gut. **(A)** Pro-tumorigenic effects of ILC3s. In a duodenum murine cancer model, NCR^+^ILC3s drive tumorigenesis by secreting IL-17 which is induced by IL-23 binding to IL-23R. In the AOM-DSS CAC model, myeloid cells regulate the IL-22 production by ILC3s in a CARD9-IL-1β-dependent, thus promoting carcinogenesis. Fungal stimulated macrophages release IL-7, which promotes IL-22 secretion from ILC3s through AhR and p-STAT3 in the *C.albicans* AOM-DSS cancer model, thus triggering CAC formation. In *H. hepaticus^+^* AOM CAC model, ILC3s-derived IL-22 promotes and maintains tumor formation through STAT3 phosphorylation in IECs. **(B)** Anti-tumorigenic effects of ILC3s. In AOM-DSS cancer model, IL-17D secreted by IECs binds to ILC3s through CD93 receptor regulating ILC3s IL-22 secretion. This shapes the colon microbiome through IECs antimicrobial peptide secretion, thus inhibiting cancer formation. Intestinal stem cells are protected from genotoxins through the AhR-IL-22 axis that regulates the DDR, avoiding the initiation of colonic cancer. In the AOM-DSS CAC model, MHC-II^+^ILC3s (CCR6^+^Lti-like cells) interact with T cells through MHC-II avoiding cancer formation. Moreover, in the AOM-DSS model, blocking the TGF-β-driven transdifferentiation of ILC3s (NKp46^+^) to ILCreg inhibits tumor growth.

Direct and indirect evidence suggest that ILC3s could play a dual role in CRC. Some studies suggest that ILC3s may contribute to intestinal carcinogenesis. In particular, it has been described that IL-22-producing ILC3s may play a crucial role in cancer related to *Helycobacter hepaticus*-induced colitis (*H.hepaticus* + AOM model). Analyses of the underlying mechanisms revealed that signal transducer and activator of transcription 3 (STAT3) phosphorylation and proliferation of epithelial cells was specifically induced by ILC3s-derived IL-22, promoting the initiation, growth and maintenance of tumors (17). Moreover, a study has shown, by using a colitis-associated cancer (CAC) model (AOM-DSS), that a myeloid cell-specific signaling protein CARD9 can regulate the IL-22 production by ILC3s during colonic inflammation, affecting the inflammation-associated carcinogenesis further supporting the pro-tumoral hypothesis. On a molecular level, IL-22 generation from ILC3s has been described to be regulated in a CARD9-IL-1β-dependent manner from myeloid cells, promoting CAC. However, T cells and NKT cells, present in the mouse model used, can also be a key source of IL-22; as a result, more research should be done to distinguish the roles of ILC3s from other IL-22-generating cells (18). Another study supporting the pro-tumoral effect of ILC3-derived IL-22 demonstrates that cytokine IL-7, generated by macrophages in response to fungal stimulation, promotes the production of IL-22 from gut ILC3s. The *Candida albicans (C. albicans)*-driven release of IL-7 from macrophages promotes aryl hydrocarbon receptor (AhR) and phospho-STAT3 (p-STAT3) binding at the IL-22 gene in ILC3s, which subsequently triggers the transcription of this cytokine for CAC formation in *C. albicans* AOM-DSS cancer model (19). Even though ILC3s are mainly studied in the colon, they are also present in other parts of the gastrointestinal tract and can be related to other types of cancer. Moreover, Crohn disease (part of IBD) is not only linked to CRC but also to CAC that can be located not only in colon but other affected areas of the small intestine (20). Apart from IL-22, IL-17 has also been linked to both roles protecting and promoting tumor growth (21). It has been demonstrated that IL-23 binds to IL-23 receptor (IL-23R) on ILC3s, which promotes the secretion of IL-17, inducing tumor development in the proximal duodenum, probably driven by NCR^+^ILC3s (IL-23R^+^Thy1^+^NKp46^+^CD3^-^) as they are expanded. Curiously, lymphoid tissue inducer cells (IL-23R^+^Thy1^+^cKIT^+^NKp46^-^CD3^-^) are decreased in tumors. Even though Th17 cells can probably also contribute to tumor development, ILC3s alone play an important role on duodenum adenomas development through IL-23 receptor signaling (22) (**Figure 1A**).

Apart from pro-tumoral effects previously mentioned, IL-22 and IL-17 have also been described to have an anti-tumoral role (23, 24). More specifically the IL-17D, which is an understudied member of the IL-17 family expressed by colonic epithelial cells, exclusively binds to ILC3s, but not T cells and may have a role in the maturation or maintenance of ILC3s function. By using the AOM-DSS cancer model, it has been determined that IL-17D binds to the CD93 receptor, regulating IL-22 secretion in ILC3s. This influences the colon microbiome, through the secretion of IEC antimicrobial peptides, thus inhibiting cancer formation (25).These results are in line with the discovery that ILC3s and gamma delta T cells (Tγδ) must produce IL-22 to effectively initiate the DNA damage response (DDR) in the intestinal epithelial stem cells in order to control mutations that lead to carcinogenesis. The secretion of IL-22 is modulated by glucosinolates (vegetable metabolites source of genotoxic stress) signaling through AhR in ILC3s and Tγδ cells (26). Therefore, intestinal stem cells are protected from genotoxic stress through the AhR-IL-22 axis that regulates the DDR avoiding the initiation of colonic cancer. Overall, these results point to an innovative homeostatic control system contrary to previously discussed papers in which enhanced IL-22 secretion acts as a booster in cancer development. Moreover, major histocompatibility complex-II+ (MHC-II+) ILC3s (CCR6^+^Lti-like cells) protect against cancer by preventing the development of invasive CRC and resistance to anti-PD-1 immunotherapy in the AOM-DSS CAC model. This is due to the interaction between ILC3s and T cells through MHC-II regulating immunological homeostasis and modifying the microbiota in a way that promotes type-1 immunity (Th1 and CD8^+^ T cells) that is lost in CRC (27) (**Figure 1B**).

It has been known that ILC3s can have plasticity towards other ILCs types and reverse (28). One study addressing plasticity during cancer development in the gut focuses on the capacity of the ILC3s to transdifferentiate into a newly discovered subpopulation of IL-10 secreting ILCs called regulatory ILCs (ILCreg) that has analog functions to Treg cells (29). It has been observed in the AOM-DSS model that ILCreg cells come from ILC3s (mostly NKp46^+^) driven by transforming growth factor beta (TGF-β) signaling that encourages tumor formation. Blockage of TGF-β pathway in ILCs disrupts ILCreg transdifferentiation and inhibits tumor growth, detecting an increased number of ILC3s in the tumor (30) (**Figure s 1A, B**).

Although pro-tumoral and anti-tumoral effects have been given to ILC3s in the progression of CRC or CAC, most of these studies have been made in mice. Regarding human studies, samples from blood were analyzed previous and after 3 months of oxaliplatin or 5FU irinotecan treatment. Compared to healthy control donors, in combined oxaliplatin-5FU irinotecan treatment, NCR^-^ILC3s (NCR^-^ILCP) were decreased whereas in mono-chemotherapy treatments NCR^+^ILC3 (NCR^+^ILCP) were decreased. These data demonstrate that chemotherapy treatments can modulate ILCs population, which can be considered in the future when ILC response and their influence in cancer are further comprehended (31). Furthermore, it has been found that infiltrating numbers of NKp44^+^ILC3s and tertiary lymphoid structures (TLS) negatively correlate with colorectal tumor progression (32), NKp44^+^ILC3s decrease whereas ILC1 and NKp44^-^ILC3s increase during tumor formation which might be indicating a plasticity from NKp44^+^ ILC3s to ILC1 (27, 32) as it has been found intermediate cellular states between the two subpopulations (27). Additionally, ILC3s were diminished in tumor tissues from CRC patients relative to adjacent normal mucosa (33), which is consistent with previously commented publications (27, 32).

## Skin cancer

**Figure 2:**
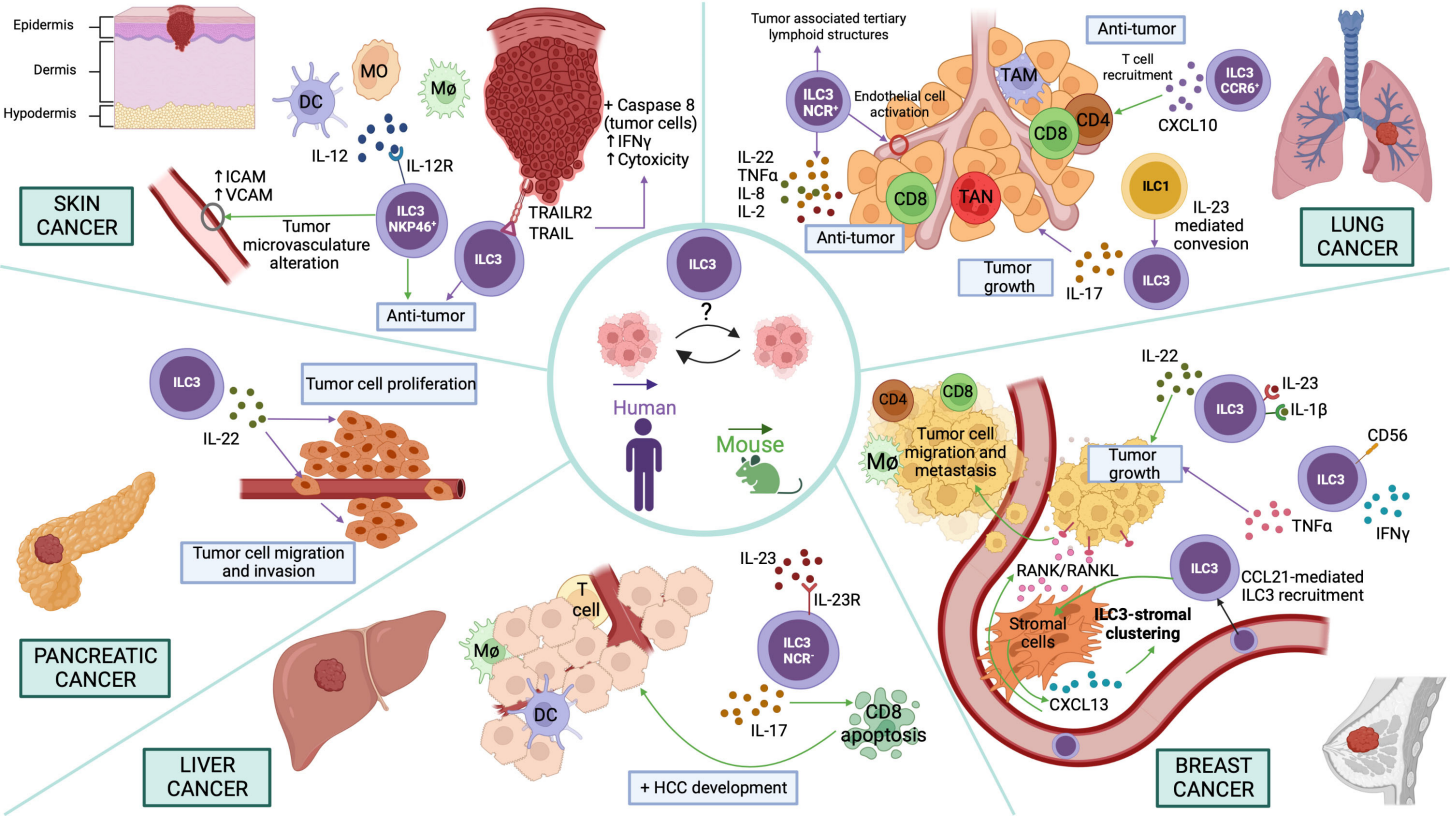
Potential functions of ILC3s in different cancer types Skin cancer. NKp46^+^ILC3s play an anti-tumor role in presence of IL-12 upregulating adhesion molecules in the vessels. ILC3s exert an anti-tumor effect through the recognition of tumor cells by TRAIL/TRAILR2, which activates Caspase-8 in target cells, IFN-γ secretion and cell cytotoxicity induction. Pancreatic cancer. ILC3s promote proliferation, invasion, and migration of pancreatic cancer cell lines *via* IL-22/IL-22R-AKT signaling. Liver cancer. NCR^-^ILC3s produce IL-23-mediated IL-17 inducing CD8^+^ T cell apoptosis and therefore promoting HCC development. Breast cancer. ILC3s secrete IL-22 promoted by IL-1β and IL-23 and thus induce tumor growth. Moreover, human CD56^+^ILC3s promote a pro-tumor immune response by inducing the production of TNF-α. Furthermore, CCL21-mediated ILC3 recruitment promotes CXCL13 secretion by stromal cells that can regulate ILC3-stromal cell clustering. Besides, CXCL13 induces RANK/RANKL signaling to promote tumor cell motility and lymph node metastases. Lung cancer. IL-23-induced conversion of ILC1 to ILC3 leads to IL-17-mediated tumor growth. In addition, NCR^+^ILC3 accumulation promotes the establishment of tumor-associated tertiary lymphoid structures. Moreover, NCR^+^ILC3s can secrete IL-22, TNF-α, IL-8 and IL-2, promoting an anti-tumoral effect in lung cancer. Furthermore, CCR6^+^ ILC3s secrete CXCL10, which facilitates CD4^+^ and CD8^+^ T lymphocyte recruitment to the tumor, thus decreasing lung cancer progression. In the figure, purple arrows indicate pathways demonstrated in studies carried out with human samples and green arrows in mouse models.

Skin cancer, which includes both malignant melanoma (MM) and non-melanoma skin cancer (NMSC), constitutes the most common malignancy in Caucasians (34). The incidence of both MM and NMSC has increased significantly over the last few decades (34). Regarding skin cancer, ILC3s showed a role in melanoma immunosurveillance, for example, *in vivo* experiments with B-16 melanoma cells demonstrated that NKp46^+^ILC3s cells (called at that moment Ltis) play a significant anti-tumor role in presence of IL-12 (35) (**Figure 2**). NKp46^+^ILC3s cells can initiate an inflammatory cascade and alter the tumor microvasculature by upregulating adhesion molecules, leading to the suppression of tumor growth. This suppression mechanism is IL-12R- and RORγt-dependent (35) (**Figure 2**).

Curiously, the tissue microenvironment has also been reported to determine the phenotype of ILC3s. ILC3s present or not the capability to inhibit the growth of IL-12-expressing melanoma tumors depending on the tissue where the ILC3s reside originally. This ability is determined by the different gene expression signatures they present, depending on the tissue resident, suggesting that the function of ILC3s is shaped not only for their lineage commitment but also by the tissue microenvironment (36). Moreover, purified human ILC3s, isolated from peripheral blood or non-cancerous human liver tissue of healthy donors, can kill human melanoma cells (SK-Mel-37) and promote anti-tumor immune response, due to the recognition of tumor cells by tumor necrosis factor-related apoptosis-inducing ligand (TRAIL)/TRAILR2, which activates Caspase-8 in target cells and interferon gamma (IFN-γ) secretion, inducing cell cytotoxicity (37) (**Figure 2**). However, in mice, it has been shown that TRAIL is expressed on mouse NK or ILC1s, and the surface expression is dependent on NKp46 (Ncr1) (38, 39). Interestingly, NKp46^+^ILC3 cells can convert into ILC1s (ex-ILC3 cells) with a concomitant decrease in RORγt and increase in T-bet and Notch signaling by a plasticity process (40, 41). Therefore, it should be verified whether human ILC3s, after being in culture, are not able to convert by plasticity into other cell types, such as ILC1s, thus presenting TRAIL on the surface.

Furthermore, NCR1 C14R mutation decreases NKp46 surface expression, inducing the destabilization of NCR1 and the accumulation of NKp46 in the endoplasmic reticulum. Ly5.1^C14R^ mice showed an increased number of early maturation stage NK cells and CD49a^+^ILC1s, which lacked TRAIL expression, and undetectable number of NKp46^+^ILC3s (42). To test whether altered NKp46 surface expression in Ly5.1^C14R^ mice modifies the ability to control tumor development, mice were inoculated with B16-F10 melanoma cells, which are known to be controlled by NK cells. This study demonstrated that surface disruption of NKp46 in Ly5.1^C14R^ mice greatly diminished *in vivo* the NK capacity to control tumor development and escape (42). Considering this study, we cannot rule out whether the observed phenotype is due to NK cells or TRAIL^+^ILC1s or whether the decreased number of Nkp46^+^ILC3s in the mouse could be also playing an important role.

## Breast cancer

Breast cancer is the most frequent malignant neoplasm in women that causes significant morbidity and mortality. It is described that the tumor microenvironment plays a relevant role in tumor growth. The association between breast cancer progression and increased IL-22 levels and ILC3s infiltrate is well determined (43, 44). Indeed, IL-22 promoted the proliferation of breast cancer cells in a STAT3-dependent manner (45). In addition, using a 4T1 breast cancer model, the authors demonstrated that ILC3s are a major source of IL-22 promoted by IL-1β and IL-23 (**Figure 2**). Thus, blocking IL-22 function could decrease breast cancer progression induced by IL-1β and IL-23 (45). Furthermore, a study performed in human lymph nodes (LN) identified a new population of ILC3s, CD56^+^ILC3s, which not only has a phenotype similar to ILC3s but also expresses cytotoxicity genes in common with NK cells. Activated CD56^+^ILC3s from donor and patient LN could acquire cytotoxic capacity producing IFN-γ or could promote a pro-tumor immune response inducing the production of tumor necrosis factor α (TNF-α) (**Figure 2**). Cytotoxic and helper ILC analysis showed a switch toward NK cells in tumor-draining lymph nodes. Thus, the local tumor microenvironment inhibited NK cell functions through downregulation of NCR, but cytokine stimulation restored their functionality (46).

Besides, *in vivo* ILC depletion mouse experiments and analysis of tumor sections from different patients showed that RORγt^+^ILC3s are associated with lymphatic tumor cell invasion and lymph node metastases in breast cancer by regulating the cancer cell chemokine profile in the tumor microenvironment (43). This study showed that ILC3s recruitment into a breast tumor model is dependent on chemokine (C-C motif) ligand 21(CCL21). These cells promoted chemokine (C-X-C motif) ligand 13 (CXCL13) secretion by stromal cells that can regulate ILC3-stromal cells clustering. In addition, CXCL13 induces receptor activator of nuclear factor κ B (RANK)/RANKL signaling to promote tumor cell motility and lymph node metastases (43) (**Figure 2**).

## Pancreatic cancer

Pancreatic cancer is one of the main causes of cancer death worldwide due to its rapid progression, early metastasis, and late diagnosis (47, 48). Recently, emerging evidence demonstrated the existence of ILCs in the pancreas and their key role in pancreatic disease and health like in other tissues (47). Analogous to other cancer types, pancreatic cancer tissues showed increased IL-22 secretion level being ILC3s the main source of this cytokine. ​​An increased frequency of ILC3s was positively correlated with the clinical features of pancreatic patients. Co-culture experiments demonstrated that ILC3s isolated from human pancreatic cancer tissues promote the proliferation, invasion, and migration of pancreatic cancer cell lines *via* IL-22/IL-22R-AKT signaling, leading to the progression of the pathology (49) (**Figure 2**).

More studies in mouse models and patient biopsies are necessary to discover and describe in depth the different ILC subsets involved in pancreatic cancer, and to characterize the interaction between these subsets and the tumor microenvironment. All these advances would be important to fully understand the pathology and design new therapies.

## Liver cancer

Liver cancer is the fifth most frequent cancer and the fourth most common cause of cancer-associated death in the world. The main types of primary liver cancers are hepatocellular carcinoma (HCC) and intrahepatic cholangiocarcinoma (ICC) (50). HCC originates from hepatocytes and is an inflammation-associated cancer. The main risk factors for the HCC development are chronic inflammatory liver diseases and liver cirrhosis (51, 52). HCC development is driven by chronic inflammation being ILC subsets, one of the early sources of cytokines in response to damage. Experiments using murine HCC models demonstrated that NCR^-^ILC3s produce IL-23-mediated IL-17, inhibiting specifically CD8^+^ T cell immunity by promoting lymphocyte apoptosis and thus inducing HCC development (51) (**Figure 2**). However, further research not only in other HCC models with different carcinogenesis conditions, but also in HCC patients are necessary to determine the expression and function of NCR^-^ILC3s and other subsets of ILC3s.

## Lung cancer

Lung cancer is the major cause of cancer-related death worldwide. Non-small-cell lung cancer (NSCLC) represents 85% of total lung cancer. Considering histology features, there are two main subtypes of NSCLSs, adenocarcinoma and squamous cell carcinoma. These subtypes show different characteristics related to biology, epidemiology and genetics (53–55). Currently, the principal therapy used in lung cancer patients is the immunotherapy. Indeed, both CTLA-4 and PD-1 blocking antibodies have been shown to be effective in lung cancer (56, 57). Interestingly, co-culture experiments of sorted human ILCs from NSCLCs and lung cancer cells demonstrated that IL-23-induced conversion of ILC1s to ILC3s leads to IL-17-mediated tumor growth and expansion in the tumor microenvironment, indicating that the IL-23/ILC3/IL-17 axis may be a potential target to treat IL23-producing lung cancers (53) (**Figure 2**). On the other hand, patient studies have demonstrated that the number of NCR^+^ILC3s is significantly increased in stage I/II NSCLC tissue compared to advanced stages of the tumor. This NCR^+^ILC3s accumulation promotes the establishment of tumor-associated TLS, which are predictors of a good prognosis (58). Moreover, *in vitro* experiments with activated NCR^+^ILC3s freshly isolated from NSCLC tissues demonstrated that these cells are involved in the production of a proinflammatory response in the tumor microenvironment through the secretion IL-22, TNF-α, IL-8 and IL-2, and the activation of endothelial cells. Therefore, NCR^+^ILC3s seem to exert an anti-tumoral role in lung cancer (58) (**Figure 2**). Furthermore, regarding the anti-tumoral role of CCR6^+^ILC3s, experiments with murine TC-1 transplantable lung cancer model demonstrated that CCL20 and IL-1β induce the CCR6^+^ILC3s recruitment and activation to the tumor. These activated CCR6^+^ILC3s can secrete CXCL10 that promotes CD4^+^ and CD8^+^ T cells migration to the tumor, enhancing anti-tumor immunity and response to immune checkpoint blockade (**Figure 2**). Thus, these data demonstrate the ability of ILC3s to turn cold tumors into hot tumors and to create a robust anti-tumor microenvironment (59).

Considering all these observations, the regulation of the activity of different subsets of ILC3s could be a possible treatment for different pathologies in combination with current therapies but more *in vivo* experiments using animal models should be performed in order to further demonstrate this promising immunotherapy.

## Discussion

With the deepening of ILC research, the function of cell subsets and their role in mucosal immunity are becoming increasingly clear. The majority of ILC3’s functions are associated with the release of cytokines, primarily IL-22 in cancer, but also IL-17 and other molecules. However, as seen in IBD not only the cytokine is important but also the concentration and timing in the cytokine release (60–62). Overall, we believe that the controversy within studies could originate from not defining the subtype of ILC3s when conducting the research and from the differences in the tumor microenvironment. Thanks to the investigation focused on the subtype of ILC3s, it can be suggested that NCR^+^ (NKp46/44^+^) are mostly anti-tumoral by secreting IL-22 in skin and lung; however, when properly stimulated, probably by the microenvironment, they can release IL-17 and become pro-tumoral in the duodenum of the small intestine. Also, presumably NCR^-^ILC3s are pro-tumoral by secreting IL-17 in hepatocarcinoma and lung cancer whereas the IL-22 produced by these NCR^-^ILC3s promotes tumor growth and metastasis in breast and pancreas. However, in colonic tissue, the function of the IL-22 produced by ILC3s is not so clear due to lack of studies that characterize the concentration of the cytokine in the microenvironment and the different subpopulations of T cells (Th17, Tγδ, NKT) and ILC3s involved in the process. On the other hand, CCR6^+^ILC3s (Lti cells) have an anti-tumoral role in chemotherapy treated lung cancer by secreting CXCL10, which is a chemoattractant of CD4^+^ and CD8^+^ T cells. Moreover, MHCII^+^CCR6^+^ILC3s (Lti cells) are also presumably anti-tumoral in CRC development, although more research is needed to support this hypothesis. Therefore, the lack of ILC3 *in vitro* lines, specific mouse models for ILC3s in different cancers, the difficulty in properly identifying ILC3 subtypes, and the low percentage present in the body makes the study of ILC3s in cancer hard. Nevertheless, further research should be done to help us reveal new mechanisms of tumor development and identify new targets for tumor treatment.

## Author contributions

AV-N, RC-G, PX and AC-A wrote and prepared the manuscript. MG-S designed the figures. All authors contributed to the article and approved the submitted version.

## Funding

The present review was supported by Ramon y Cajal Program (RYC-2017-21837) and the grant N° RTI2018-093647-B-I00 and PID2021-122780OB-I00 to AC-A from the Ministerio de Ciencia, Innovación e Universidades (MCIU), Agenda Estatal de Investigación (AEI), with co-funding by the European Regional Development Fund (ERDF) “A way to build Europe”. AV-N is a recipient of an FPI fellowship (PRE2019-090341) from the Spanish Ministry of Science, Innovation, and Universities. RC-G is supported by “Ayudas Margarita Salas para la Formación de Jóvenes Doctores” - Universidad Autónoma de Madrid (CA1/RSUE/2021–00577) from the Spanish Ministry of Universities.

## Acknowledgments

Figures were created using BioRender.

## Conflict of interest

The authors declare that the research was conducted in the absence of any commercial or financial relationships that could be construed as a potential conflict of interest.

The handling editor declared a shared affiliation with the authors at the time of review.

## Publisher’s note

All claims expressed in this article are solely those of the authors and do not necessarily represent those of their affiliated organizations, or those of the publisher, the editors and the reviewers. Any product that may be evaluated in this article, or claim that may be made by its manufacturer, is not guaranteed or endorsed by the publisher.
